# Precision Oncology in Thymic Epithelial Tumors: Therapeutic Horizons and Implementation Barriers

**DOI:** 10.3390/ijms27156613

**Published:** 2026-07-24

**Authors:** Kübra Canaslan, Özge Yetginoğlu, Yasuhiro Tsutani, Aparna Sharma, Daniel E. Mansila, Amirhossein Emami, Hassan Abolhassani, Fatemeh Ardeshir-Larijani

**Affiliations:** 1 Department of Medical Oncology, Mehmet Akif İnan Research and Training Hospital, Şanlıurfa 63040, Türkiye; 2 Department of Medical Oncology, Dokuz Eylül University, İzmir 35330, Türkiye; 3 Division of Thoracic Surgery, Kindai University Faculty of Medicine, Osaka 589-0014, Japan; 4 Department of Medical Oncology, National Cancer Institute (Jhajjar)-AIIMS, New Delhi 124105, India; 5 Department of Thoracic Surgery, Instituto de Oncologia Angel H. Roffo, Buenos Aires C1417DTB, Argentina; 6 Department of Medicine, Cancer Institute, Tehran University of Medical Sciences (TUMS), Tehran 1416634793, Iran; 7 Clinic for Pediatric Immunology and Rheumatology, Center for Pediatrics and Adolescent Medicine, University Hospital Bonn, 53127 Bonn, Germany; 8 Research Center for Immunodeficiencies, Pediatrics Center of Excellence, Children’s Medical Center, Tehran University of Medical Sciences, Tehran 1419733151, Iran; 9 Research Institute for Oncology/Hematology and Cell Therapy (RIOHCT), Tehran University of Medical Sciences (TUMS), Tehran 1411713135, Iran

**Keywords:** thymic epithelial tumors, precision oncology, genomic alterations, antibody–drug conjugates, targeted therapy, ctDNA

## Abstract

Thymic epithelial tumors (TETs), comprising thymomas and thymic carcinomas, are rare, biologically heterogeneous thoracic malignancies with limited therapeutic advances since platinum-based regimens were adopted decades ago. Recent genomic and immunophenotypic profiling has uncovered recurrent genomic alterations and high programmed death-ligand 1 (PD-L1) expression, particularly in thymic carcinoma, yet few targeted or immunotherapies have achieved regulatory approval. This mini-review synthesizes current knowledge on TET heterogeneity and the genomic landscape, evaluates the clinical evidence supporting targeted agents and immune checkpoint inhibitors, and examines emerging biomarkers, including circulating tumor DNA. We also address practical barriers to precision oncology in TETs: challenges of next-generation sequencing implementation and cost in resource-limited settings; scarcity of large, biomarker-driven trials; and safety concerns unique to TETs (immune-related toxicity). Finally, we discuss the translational hurdles for antibody–drug conjugates and cellular therapies, limited validated cell surface targets, antigen heterogeneity, and preclinical model gaps and outline strategic paths forward to enable rational, safe, and equitable precision therapeutics for TET patients.

## 1. Background

The thymus is an immune organ located in the anterior mediastinum and plays an essential role in T-cell maturation and selection. Thymic epithelial tumors (TETs) are rare malignancies, with reported incidence rates ranging from approximately 0.13 to 0.32 per 100,000 person-years across population-based cohorts. Geographic variation has been reported, with higher incidence rates in East Asian populations and lower rates in some African populations [1,2,3]. TETs arise from thymic epithelial cells and include thymomas, thymic carcinomas (TCs), and thymic neuroendocrine tumors [4]. Despite their shared origin, these entities differ markedly in biology, clinical behavior, and prognosis.

**Figure 1:**
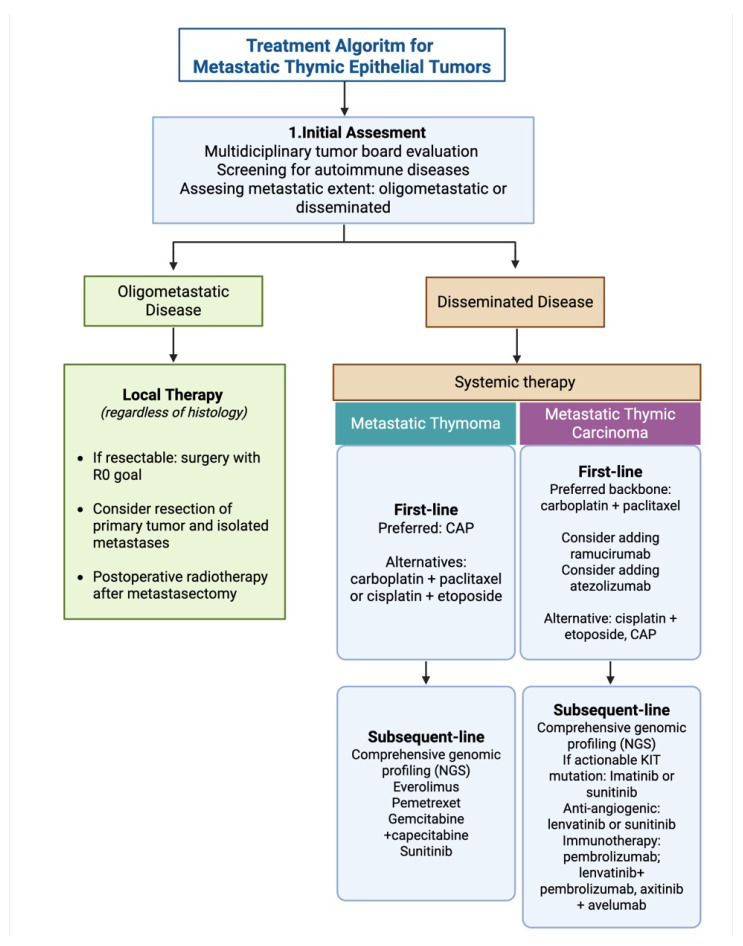
Current landscape of treatment of metastatic TETs. The algorithm outlines a disease extent- and histology-based approach to metastatic thymic epithelial tumors according to current National Comprehensive Cancer Network (NCCN) and European Society for Medical Oncology (ESMO) guideline recommendations. Initial evaluation includes multidisciplinary assessment, autoimmune disease screening, and classification as oligometastatic or disseminated disease. Oligometastatic disease may be managed with local therapies when feasible, whereas disseminated disease requires systemic treatment selected according to histology. CAP-based therapy is commonly preferred for metastatic thymoma, while carboplatin plus paclitaxel is a preferred backbone for metastatic thymic carcinoma, with selected use of ramucirumab, immunotherapy, antiangiogenic therapy, targeted therapy, and genomic profiling in subsequent-line settings. CAP, cyclophosphamide, doxorubicin, and cisplatin; NGS, next-generation sequencing. Treatment options were summarized based on NCCN-2026 [9] and ESMO practice guideline [10,11] guideline recommendations [9,10]. Created in BioRender. Canaslan, K. (2026). https://BioRender.com/903go6z accessed on 19 July 2026.

Platinum-based chemotherapy remains the standard first-line systemic treatment for advanced or unresectable TETs. In TC, recent data support the addition of antiangiogenic inhibitors (vascular endothelial growth factor receptor 2 (VEGFR2) monoclonal antibody, ramucirumab) to carboplatin and paclitaxel. However, unlike lung cancer, in which next-generation sequencing (NGS) and immunotherapy have reshaped treatment, therapeutic progress in TETs has been limited. No molecularly targeted therapy is currently approved specifically for TETs by the United States Food and Drug Administration (FDA). Nevertheless, agents such as multikinase inhibitor lenvatinib [5], mechanistic target of rapamycin (mTOR) inhibitor everolimus [6], and KIT inhibitor imatinib [7] have demonstrated clinical activity in selected settings. The current systemic treatment approach for metastatic TETs is summarized in Figure 1.

The development of individualized therapy in TETs is constrained by disease rarity, histologic heterogeneity, low frequency of canonical actionable drivers, limited prospective clinical trials, immune-related toxicity, and unequal access to molecular testing and off-label therapies [8]. Therefore, the central challenge is not simply identifying molecular abnormalities but demonstrating that biomarker-directed treatment improves clinically meaningful outcomes.

This review focuses on the gap between biological target identification and clinical actionability in TETs. It examines why frequently detected molecular alterations or protein expression do not consistently predict treatment benefit. It also addresses the practical implementation of precision oncology, including genomic testing and cost-related barriers. Emerging biomarkers and imaging-based technologies are also evaluated in the context of perioperative decision-making and postoperative surveillance.

## 2. Biological and Clinical Heterogeneity of Thymic Epithelial Tumors

TETs represent a spectrum of diseases characterized by histologic and clinical heterogeneity [12]. The World Health Organization (WHO) classifies thymomas (types A, AB, B1, B2, and B3) based on epithelial cell morphology and the proportion of non-tumoral lymphocytes. In contrast to thymomas, TCs are more aggressive, lack an organoid architecture, and closely resemble extrathymic squamous cell carcinomas [4,13].

This heterogeneity extends beyond conventional histologic classification. A recent integrative genomic and transcriptomic analysis classified TETs into three molecular groups: *GTF2I*-mutant thymomas, copy-number-altered thymomas characterized by Insulin Receptor Substrate 4 (IRS4) activation, and thymic carcinomas. These groups displayed distinct transcriptional programs, immune phenotypes, and putative cells of origin, indicating that histologic classification alone may not fully capture the biological diversity of TETs [14]. Although this framework may support future biomarker development and patient stratification, its predictive value for treatment selection remains to be validated.

These histologic and molecular differences have direct clinical implications. Thymomas are often more indolent and are frequently associated with autoimmune paraneoplastic disorders, especially myasthenia gravis, pure red cell aplasia, and Good’s syndrome (associated with hypogammaglobulinemia and phenocopies of inborn errors of immunity). In contrast, TC is generally more aggressive, more likely to metastasize early, less frequently associated with autoimmunity, and associated with poorer survival [15].

Recent multi-omics clustering has further dissected TC into immunologically “hot” and “cold” subsets [16]. The “hot” TC cluster exhibits significantly higher CD3+ and CD4+ T-cell infiltration, which translates clinically into a longer median overall survival (OS) and higher progression-free survival (PFS) ratios when treated with immune checkpoint inhibitors (ICIs) compared to the “cold” TC subset [16]. Additionally, programmed death-ligand 1 (PD-L1) [17,18] and lymphocyte-activation gene 3 (LAG-3) [12] were reported to be highly expressed in single-cell analyses, and both are known targets for immunotherapy.

## 3. Genomic Landscape of Thymic Epithelial Tumors

Tumor mutational burden (TMB) varies according to TET histology. In the The Cancer Genome Atlas (TCGA) cohort, the overall mean TMB was low at 0.48 mutations/Mb; however, the cohort was predominantly composed of thymomas, and histology-stratified analysis demonstrated significantly higher TMB in TC than in thymoma subtypes [19]. Despite this, comprehensive genomic profiling has elucidated distinct molecular drivers between subtypes. The indolent thymomas are characterized primarily by point mutations in *GTF2I*, most commonly p.L424H or p.L404H, depending on transcript annotation. These variants affect the second conserved general transcription factor II-I (TFII-I) repeat domain near the DNA-binding region [20]. These alterations have been reported in up to 58% of tested thymoma cohorts, appear highly specific to thymoma, and are considered thymoma-associated genomic alteration [21].

TC harbors distinct mutational profiles involving tumor suppressors and epigenetic regulators such as *TP53* (30–36%), *CDKN2A/B*, *BAP1*, *CYLD*, and *SETD2* [16,22,23,24,25]. In a recent comprehensive profiling cohort, alterations were identified in cell-cycle-related genes in 13% of cases, DNA repair pathways in 8%, and chromatin-remodeling genes in 19%. A distinct subset of cases (8%) demonstrated mismatch repair deficiency (dMMR) or high microsatellite instability (MSI-H) status [25]. Furthermore, TCs demonstrate significantly higher frequencies of gene amplifications in *ERBB2*, *MDM4*, and *MYC* [16,23].

While immunohistochemical overexpression of the c-KIT protein is observed in only 2% to 4% of thymomas, it is a hallmark feature detected in 46% to 86% of TC cases [23,26]. However, despite this widespread and strong protein-level overexpression, true gain-of-function *KIT* gene variants (e.g., p.V560del and p.L576P in exon 11, p.D820E in exon 17) are found in only approximately 7 to 11% of TCs [19,27].

Precision oncology studies have identified pathogenic germline variants in up to 18% of patients with advanced TETs, particularly in DNA damage repair-associated genes such as *BRCA1/2*, *BAP1*, *MUTYH*, and *CHEK2* [16,28]. This high rate of pathogenic germline variants (PGVs) leads to Homologous Recombination Deficiency (HRD) signatures in up to 53% of patients, uncovering vulnerabilities that could potentially be exploited by poly ADP-ribose polymerase (PARP) inhibitors [16].

## 4. Targeted Therapy in TETs: Clinical Evidence and Limitations

**Figure 2:**
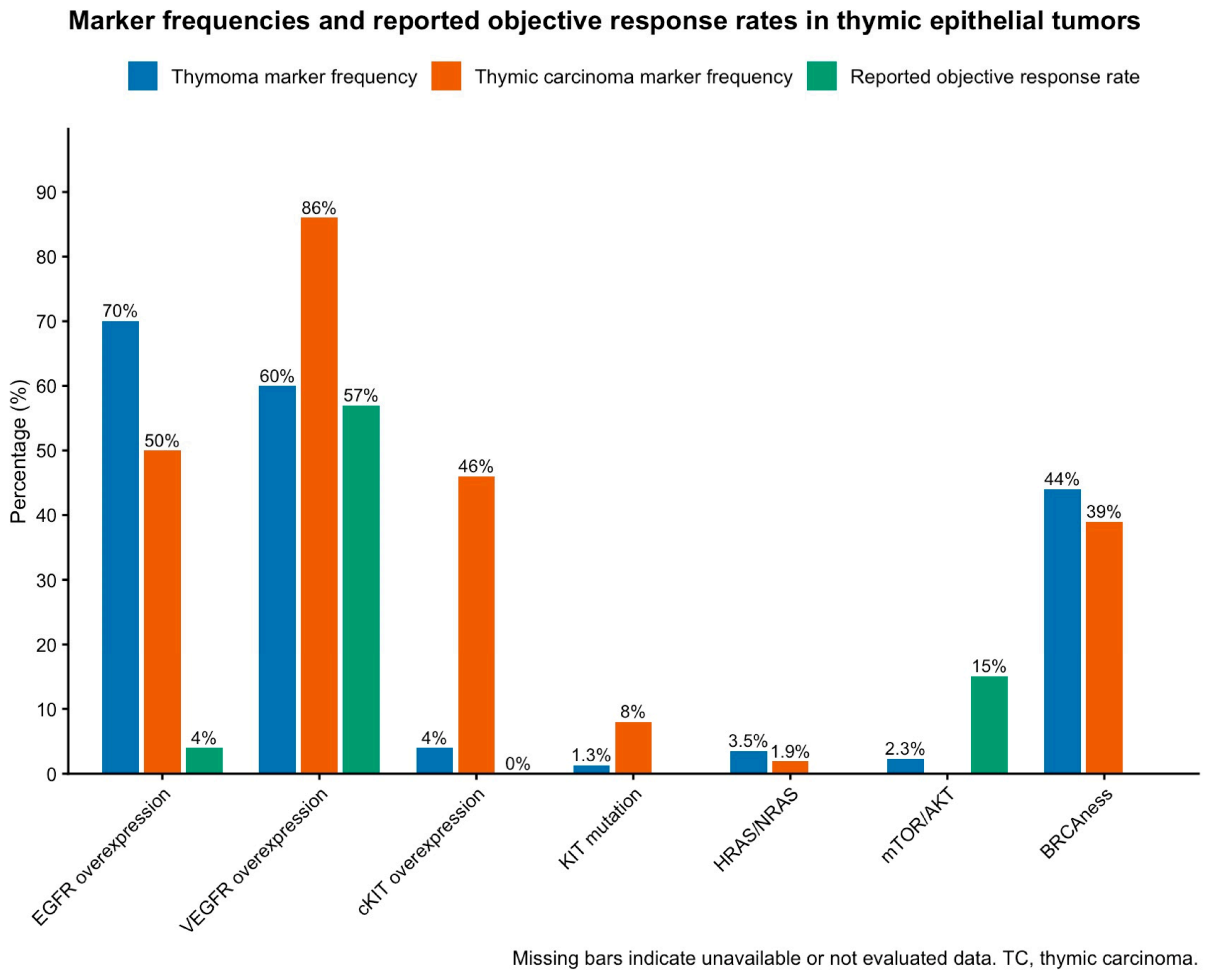
Comparative frequencies of selected molecular markers and reported objective response rates in thymic epithelial tumors. The bar plot compares selected target expression or genomic alteration frequencies in thymoma and thymic carcinoma with reported objective response rates (ORRs) for corresponding targeted or pathway-directed therapeutic strategies. ORR was defined as the proportion of patients achieving a complete or partial response. For each marker, the plotted alteration or expression frequency represents the highest reported value from the cited published study or review. EGFR [29], VEGFR [30], and cKIT [31] represent protein overexpression or pathway-level target expression, whereas KIT mutation [23], HRAS/NRAS [23], mTOR/AKT [23], and BRCAness [16] represent genomic or pathway alterations. ORRs were derived from clinical studies evaluating the corresponding therapeutic class, including anti-EGFR therapy [32], antiangiogenic therapy [33], KIT-directed therapy [7,34], and mTOR inhibition [6], where available. Missing bars indicate unavailable or unevaluated data. This figure was independently created based entirely on aggregate data extracted from previously published studies and reviews; it does not include unpublished original data and was not reproduced or adapted from any single publication. ORR, objective response rate; TC, thymic carcinoma. Created in BioRender. Canaslan, K. (2026). https://BioRender.com/4a2tfw9 accessed on 19 July 2026.

While cytotoxic chemotherapy remains the standard frontline treatment for advanced TETs [10], precision oncology has paved the way for evaluating targeted agents. However, translating molecular discoveries into clinical efficacy has proven challenging, yielding mixed results that are heavily dependent on the specific pathway being targeted (Figure 2). The main targeted agents evaluated in clinical studies of TETs are summarized in Table 1.

### 4.1. Antiangiogenic Strategies

Currently, multi-kinase inhibitors, mainly tyrosine kinase inhibitors (TKIs), with potent anti-angiogenic properties represent the most clinically active class of targeted drugs for refractory tumors [35,36]. Their biological rationale is supported by vascular endothelial growth factor (VEGF) overexpression in 86–88% of TETs and high VEGFR2 expression in 88.5% of aggressive subtypes, including TC and type B3 thymoma [30].

In a phase II trial of heavily pretreated patients, sunitinib yielded an objective response rate (ORR) of 26% and disease control rate (DCR) of 91% in the TC cohort, establishing it as an off-label later-line option [37]. In the multicenter phase II REMORA trial, lenvatinib demonstrated an ORR of 38% and a median PFS of 9.3 months in previously treated TC, leading to its recognition as a highly promising salvage therapy [38]. Long-term follow-up of REMORA further supported the durability of lenvatinib activity, with a median OS of 28.3 months and a 36-month OS rate of 35.7% [5]. Although lenvatinib has not received FDA or European Medicines Agency (EMA) approval specifically for TC, it is approved in Japan for unresectable TC and it has been recommended in NCCN guideline [39].

Moving anti-angiogenics to the frontline, the phase II RELEVENT trial recently evaluated the VEGFR2 monoclonal antibody ramucirumab combined with carboplatin and paclitaxel in treatment-naive advanced TC. The ORR was 80% by investigator assessment and 57.6% by central review, with a median PFS of 18.1 months. These results indicate substantial first-line activity, although confirmation in randomized studies is required [33].

### 4.2. mTOR, AKT, and PI3K Pathway Inhibitors

Given the frequent activation of the PI3K/mTOR pathway, the mTOR inhibitor everolimus was evaluated in a phase II trial. Everolimus achieved an overall DCR of 88%, with a median PFS of 16.6 months in thymoma and 5.6 months in TC. However, this efficacy was counterbalanced by a high incidence of severe toxicities, including a 6% incidence of fatal pneumonitis [6]. The pan-PI3K inhibitor buparlisib showed only modest activity with a 7.1% ORR while carrying substantial toxicities [40].

### 4.3. EGFR and KIT: Lesson in Target Selection

One of the most notable disappointments in targeted therapy involves the epidermal growth factor receptor (EGFR) pathway. EGFR protein is frequently overexpressed, found in approximately 70% of thymomas and 50% of TCs [29,35,36]. Despite this robust and widespread overexpression, anti-EGFR targeted therapies have been largely unsuccessful. In a phase II trial evaluating the EGFR-TKI gefitinib in heavily pretreated patients, the ORR was a 4%, with no complete responses [32]. Similarly, a phase II trial combining the EGFR-TKI erlotinib with bevacizumab yielded no objective responses, leaving 60% of patients with stable disease and 40% with progressive disease as their best response [41]. The primary reason for this clinical failure is that EGFR overexpression almost never correlates with activating *EGFR* gene variants, which are the actual biological predictors of sensitivity to EGFR-directed TKI therapy [42].

A similar paradigm is observed with the c-KIT expression and KIT mutations. Early phase II trials testing the KIT inhibitor imatinib in unselected cohorts based on c-KIT overexpression reported no objective responses [7,34]. However, in TCs with activating somatic KIT variants such as exon 11 (p.V560del), patients have demonstrated impressive and occasionally long-lasting tumor shrinkage with imatinib [43,44]. Therefore, routine screening for c-KIT followed by mutational sequencing is critical to identify the minority of patients who may benefit from KIT inhibitors. As a result, EGFR overexpression and c-KIT overexpression without pathogenic gain-of-function mutation should be considered non-predictive.

### 4.4. Somatostatin Receptor-Targeted Therapy

Immunohistochemical evaluations demonstrate that approximately 78% of TETs express at least one somatostatin receptor (SSTR) subtype, predominantly expressing SSTR2A on malignant neoplastic epithelial cells and SSTR3 on infiltrating reactive thymocytes [45]. This high level of expression provides a biological rationale for utilizing somatostatin analogs, such as octreotide, particularly in pretreated patients whose tumors exhibit positive uptake on SSTR-based functional imaging [46,47].

Octreotide monotherapy demonstrated modest clinical activity, yielding an ORR of 10.5% and a median PFS of 2.0 months. However, the subsequent addition of prednisone was associated with an ORR of 31.6% and a median PFS of 9.2 months [46]. Activity appeared to differ by histology: responses were observed primarily in thymoma, whereas no objective responses were reported in patients with TC. The combination therapy requires strict safety monitoring. However, prolonged high-dose corticosteroid treatment may worsen thymoma-associated immunodeficiency and increase the risk of infection. [46]. Therefore, while SSTR-targeted therapy offers a viable chemo-free alternative, its efficacy is largely confined to thymomas and must be balanced against the risk of toxicity.

### 4.5. IGF-1R, Histone Deacetylase, and DNA Damage Response Strategies

Cell-cycle inhibition has been evaluated with the cyclin-dependent kinases 4 and 6 (CDK4/6) inhibitor palbociclib. In a phase II trial of recurrent or refractory advanced TETs, palbociclib achieved an ORR of 12.5% and a median PFS of 11.0 months [48]. Trials targeting the insulin-like growth factor 1 receptor (IGF-1R) pathway with the monoclonal antibody cixutumumab showed a 14% response rate in thymomas, but no responses in TC. New or worsening autoimmune disorders occurred in 24% of patients, limiting its clinical use [49]. Belinostat, a Histone Deacetylase (HDAC) inhibitor, has shown limited objective activity in recurrent or refractory TETs, with disease stabilization in some patients [50].

**Table 1:** Targeted therapies with clinical evaluation in thymic epithelial tumors.

Agent	Target/Class	TET Population	Evidence	Efficacy	Key Limitation
Gefitinib [32]	EGFR tyrosine kinase inhibitor	Previously treated TC and thymoma	Phase II	ORR 4%	Poor efficacy due to the rarity of EGFR mutations
Erlotinib + bevacizumab [41]	EGFR tyrosine kinase inhibitor + VEGF inhibitor	Previously treated TC and thymoma	Phase II	ORR 0%	Lack of efficacy and high rate of toxicity
Ramucirumab + carboplatin/paclitaxel [33]	VEGFR2 monoclonal antibody + chemotherapy	Treatment-naive advanced TC	Phase II	ORR 57%, median PFS 18.1 months	High rate of grade ≥ 3 AEs, excludes patients with untreated brain metastases
Lenvatinib [38]	VEGFR/FGFR/RET/KIT multikinase inhibitor	Previously treated TC	Phase II	ORR 38%, median PFS 9.3 months	High toxicity profile requiring dose modifications (reductions needed in 100% of patients)
Sunitinib [37]	VEGFR/KIT/PDGFR multikinase inhibitor	Refractory TC + thymoma	Phase II	ORR 26% in TC and 6% in thymoma	High toxicity (70% grade 3/4 events) and minimal activity in thymoma
Everolimus [6]	mTOR inhibitor	Previously treated thymoma and TC	Phase II	ORR 7% in TC and 9% in thymoma	70% dose interruptions, high risk of grade ≥ 3 pneumonitis
Imatinib [7,34]	KIT/PDGFR inhibitor	Mostly TC/B3 thymoma	Phase II/small studies	0% in unselected patients, rare PR in KIT-mutated tumors	Lack of efficacy even in KIT-mutated TETs
Cixutumumab [49]	IGF-1R antibody	Recurrent thymoma and TC	Phase II	ORR 0% in TC, 14% in thymoma	Induced new or worsened existing autoimmune diseases in thymoma, no objective activity in TC
Belinostat [50]	HDAC inhibitor	Recurrent/refractory TET	Phase II	ORR 0% in TC, 8% in thymoma	Lack of efficacy
Palbociclib [48]	CDK4/6 inhibitor	Previously treated thymoma and TC	Phase II	ORR 12%, median PFS 11 months	Hematologic toxicity (grade 3 neutropenia, 41%) requiring dose modifications
Milciclib [51]	CDK/Src-family kinase inhibitor	B3 thymoma and TC	Phase II	ORR 3%, median PFS 5 months	Lack of efficacy
Buparlisib [40]	PI3K inhibitor	Relapsed/refractory thymoma	Phase II	ORR 7%, median PFS 11 months	Grade 3–4 adverse events (14–21%)

AE: adverse events; ORR, objective response rate; PR, partial response; PFS, progression-free survival; TC, thymic carcinoma; TET, thymic epithelial tumor; EGFR, epidermal growth factor receptor; VEGF/VEGFR, vascular endothelial growth factor/receptor; FGFR, fibroblast growth factor receptor; RET, rearranged during transfection; KIT, KIT proto-oncogene receptor tyrosine kinase; PDGFR, platelet-derived growth factor receptor; mTOR, mechanistic target of rapamycin; IGF-1R, insulin-like growth factor 1 receptor; HDAC, histone deacetylase; CDK, cyclin-dependent kinase; PI3K, phosphoinositide 3-kinase.

## 5. Immune Checkpoint Inhibition: Efficacy Versus Autoimmune Toxicity

Immunotherapy in TETs is primarily focused on immune checkpoint inhibitors (ICIs). Phase II trials of pembrolizumab (anti-PD-1) monotherapy have demonstrated ORRs of approximately 19.2–22.5% in pretreated TC and 28.6% in thymoma, providing durable clinical benefits for a subset of patients [52,53]. Results with nivolumab (anti-PD-1) have been less consistent. The NIVOTHYM trial reported an ORR of approximately 12% in a mixed cohort of patients with type B3 thymoma and TC, whereas no objective responses were observed in the PRIMER trial of unselected patients with recurrent TC [54].

Despite these encouraging signals, immunotherapy in TETs is called a “double-edged sword” due to a prohibitive risk of severe immune-related adverse events (irAEs) [55,56]. Because the thymus is the central organ for T-cell maturation and immune tolerance, ICIs can trigger life-threatening systemic autoimmunity. In thymoma, disrupted thymic architecture, reduced autoimmune regulator (AIRE)-dependent self-antigen expression, defective negative selection, and impaired regulatory T-cell generation allow autoreactive T cells to escape into the circulation. PD-1/PD-L1 or cytotoxic T-lymphocyte-associated protein 4 (CTLA-4) blockade may weaken residual peripheral tolerance and activate these pre-existing autoreactive clones, leading to autoantibody production and T-cell-mediated tissue injury [57]. Accordingly, irAE rates are substantially higher in thymoma than in TC, reaching 71.6% and 15.4%, respectively. Severe manifestations include myocarditis, polymyositis, myasthenia gravis, and hepatitis, which may require treatment discontinuation and can be fatal [36,56,58]. Consequently, ICIs are generally discouraged in thymoma and require rigorous monitoring in TC. To enhance efficacy while managing toxicity, current research is shifting toward combinatorial strategies, such as combining ICIs with anti-angiogenic TKI agents like axitinib (CAVEATT trial, 34% ORR) or lenvatinib (PECATI trial) and integrating ICIs with frontline platinum-based chemotherapy [13,59,60]. The key immune checkpoint inhibitor studies in TETs, including single-agent PD-1/PD-L1 blockade and antiangiogenic–immunotherapy combinations, are summarized in Table 2.

## 6. Antibody-Drug Conjugate Therapy, Vaccines, and Cellular Therapy

The development of antibody–drug conjugates (ADCs) and antigen-directed cellular therapies depends on identifying cell-surface targets that are widely expressed on tumor cells but absent or minimally expressed in normal tissues [18,69]. In rare malignancies like TETs, the discovery of such targeted cell surface antigens has been limited, which has delayed the clinical translation of these advanced therapeutic modalities. Recent comprehensive tissue microarray studies have evaluated various potential surface targets, such as HER2, PSMA, ROS1, and ALK, which have demonstrated little or no expression in most TETs [18]. In contrast, trophoblast cell-surface antigen 2 (Trop-2) demonstrated consistently high membranous expression across all histologic subtypes of TETs and has emerged as a promising therapeutic target [18]. Broader surface-antigen profiling has also identified high expression of additional candidate targets, including NECTIN-4 and mesothelin [25]. This robust expression of Trop-2 has paved the way for clinical trials evaluating sacituzumab govitecan, an anti-Trop-2 ADC, in patients with advanced TETs [67].

Mesothelin, another cell-surface antigen typically found on cells lining the pleura and pericardium, is frequently and strongly expressed in TC but is less common in thymoma [70]. This expression profile has led to clinical trials exploring anetumab ravtansine, a mesothelin-directed ADC, specifically in patients with TC [68]. Furthermore, the expression of mesothelin provides a strong biological rationale for the future development and evaluation of mesothelin-directed chimeric antigen receptor T cell (CAR-T cell) therapies for this patient population [71]. Another novel ADC targeting the B7-H3 surface antigen is also currently being investigated in early-phase clinical trials that include TC patients [72].

CD70 represents another appealing target for cellular therapies. This transmembrane protein contributes to B- and T-cell interactions and has limited expression in most normal tissues [73]. Clinical development of CD70-directed cellular products, including CTX130, has primarily focused on hematologic malignancies [66,74,75] and renal cell carcinoma [76]. The high prevalence of CD70 expression supports its investigation in TC, although no CD70-directed cellular therapy has yet demonstrated clinical efficacy in TETs.

The Wilms’ tumor 1 (WT1) protein is responsible for promoting oncological development and tumor vascularization and is overexpressed in approximately 80–85% of both thymomas and TCs [66]. A phase II clinical trial evaluating a WT1 peptide vaccine in advanced, pretreated patients demonstrated an ability to induce WT1-specific immune responses. However, reflecting the unique immunological risks associated with the thymus, some thymoma patients developed severe irAEs, including pure red cell aplasia and myasthenia gravis [66]. Antibody-based and vaccine-based strategies are summarized in Table 2.

## 7. Therapeutic Strategies for Autoimmune Diseases Associated with TET

Autoimmunity is one of the defining common features of TETs and reflects disruption of thymus-mediated central immune tolerance. Under physiological conditions, medullary thymic epithelial cells, major histocompatibility complex (MHC)-dependent antigen presentation, and AIRE-mediated expression of tissue-restricted antigens eliminate autoreactive T cells. In thymoma, architectural disorganization, reduced AIRE expression, impaired antigen presentation, and abnormal thymocyte selection allow autoreactive T-cell escape, contributing to autoimmune manifestations such as myasthenia gravis, pure red cell aplasia, and other immune-mediated disorders [77,78,79,80].

Recent transcriptomic and spatial profiling studies further suggest that thymoma-associated autoimmunity is sustained by abnormal medullary niches, ectopic germinal center formation, autoreactive B-cell responses, and reduced regulatory T-cell populations. This immune dysregulation may persist even after thymectomy [81,82,83,84,85,86].

Management depends on the specific autoimmune manifestation and requires multidisciplinary assessment. Thymectomy remains central to oncologic treatment when feasible and may improve some associated autoimmune disorders, although it does not uniformly restore immune tolerance. Corticosteroids and steroid-sparing agents, including azathioprine, mycophenolate mofetil, tacrolimus, and cyclosporine, remain standard options. In refractory antibody-mediated disease cases, targeted immune therapies including rituximab, complement inhibitors (eculizumab, ravulizumab, and zilucoplan), and neonatal Fc receptor (FcRn) antagonists (efgartigimod, rozanolixizumab, and nipocalimab) may be considered. Other emerging approaches, including cytokine-directed therapies targeting interleukin (IL)-6, IL-17, or IL-23 and experimental cellular strategies such as CAR-T therapy, remain investigational in TET-associated autoimmunity [83,87,88].

From an oncologic standpoint, the presence of underlying autoimmunity plays a key role in guiding treatment decisions. ICIs, while effective in some cancers, can provoke severe or even life-threatening immune-related adverse events in patients with TETs, particularly those with thymoma. For this reason, their use should be approached with great caution, especially in individuals with active or prior autoimmune disease. Overall, careful assessment of autoimmune status is essential when tailoring treatment strategies for patients with TETs.

## 8. Liquid Biopsy, Tumor Microenvironment, and ctDNA-Based Monitoring

### 8.1. Liquid Biopsy and ctDNA-Based Monitoring

Liquid biopsy techniques, including cell-free DNA (cfDNA) and circulating tumor DNA (ctDNA), are emerging as complementary tools to address the limitations of single-site tissue biopsies. The incorporation of liquid biopsy (ctDNA analysis) into the clinical workflow holds substantial promise. These tools not only enhance diagnostic accuracy and subtyping but also provide a mechanism to identify actionable alterations, capture molecular heterogeneity, and enable longitudinal assessment of treatment response or resistance. Thereby expanding therapeutic options and enabling more targeted, personalized treatment, particularly for patients in later lines of therapy [89,90]. In prospective TET cohorts, cfDNA levels are significantly higher in TCs compared to thymomas and healthy controls [90]. This disparity likely reflects histology-specific ctDNA shedding. Aggressive TCs, with greater metastatic potential, generally show increased tumor DNA shedding into circulation, whereas indolent thymomas may have lower tumor burden and reduced plasma ctDNA detectability [91]. Consequently, cfDNA levels correlate with metastatic burden rather than primary tumor volume or local stage [22,90]. However, because the current evidence remains limited, a negative ctDNA result should not be considered sufficient to exclude residual disease or recurrence, especially in thymoma or low-volume disease.

ctDNA assays have identified tumor-associated alterations (TP53, CYLD, GTF2I) that mirror those found in primary tumor tissues [91]. Longitudinal ctDNA monitoring has shown the potential to detect minimal residual disease (MRD) following surgery [92]. In reported clinical instances, ctDNA analysis tracked a decline in tumor-specific mutations post-resection and flagged molecular recurrence a month before radiographic progression became visible [90,93]. Nevertheless, these findings remain preliminary, and ctDNA has not yet been validated for routine surveillance, postoperative decision-making, or treatment selection in TETs. Furthermore, complementary platforms utilizing Circulating Tumor Cell (CTC)-derived organoids have demonstrated high sensitivity in matching ex vivo drug sensitivity to actual patient clinical responses, laying the groundwork for real-time therapeutic monitoring [94].

### 8.2. Tumor Microenvironment and Spatial Profiling

Single-cell RNA sequencing and spatial profiling techniques, including high-resolution spatial transcriptomics and multiplex immunofluorescence, can characterize the cellular composition and interactions within the TET microenvironment [82]. In myasthenia gravis-associated thymomas, spatial analyses have localized polygenic risk signals and disease-specific ectopic germinal centers predominantly to expanded medullary regions. Mapping these physical interactions offers critical clues for treatment, distinguishing between therapy-responsive “hot” microenvironments enriched with tertiary lymphoid structures and non-responsive tumors that exhibit localized thymic epithelial cell (TEC)-driven immunosuppression [82].

Spatial profiling recently demonstrated that major pathological responses to neoadjuvant immunochemotherapy in TC are closely associated with distinct tumor microenvironments. Tumors that respond well are characterized by “hot” microenvironments with extensive immune infiltration and tertiary lymphoid structure (TLS) formation, whereas non-responsive tumors exhibit localized immunosuppression driven by TECs that exhaust infiltrating CD8+ T cells [95]. These findings may contribute to future biomarker-based patient selection and identification of mechanisms of treatment resistance, although prospective validation is required.

### 8.3. Perioperative and Surgical Implications of Emerging Precision Technologies

In TETs, multidisciplinary treatment decisions are strongly influenced by resectability, histologic subtype, local invasion, completeness of resection, and postoperative recurrence risk [9]. Therefore, ctDNA, radiomics, and digital pathology should not be viewed only as tools for systemic treatment selection but also as potential adjuncts for surgical planning and postoperative surveillance. Preoperatively, computed tomography (CT) remains the reference imaging modality for evaluating disease extent, response to induction therapy, and potential resectability after systemic treatment [96]. In this context, radiomics may provide additional quantitative information beyond conventional imaging. CT-based radiomic models have shown potential for predicting resectability and tumor–node–metastasis (TNM) stage in TETs [97]. Additionally, magnetic resonance imaging (MRI)-based radiomics has been explored for predicting pathologic classification and staging [98]. However, radiomics remains investigational and should be considered complementary to expert radiologic assessment rather than a replacement for multidisciplinary review [99].

Induction treatment followed by surgery has produced encouraging complete resection rates in initially unresectable TETs, and pathologic response and completeness of resection have been associated with prognosis [100]. Standardized assessment of viable tumor and treatment-related changes is therefore important for postoperative risk stratification and clinical trial design [101]. Digital pathology may improve the reproducibility of these assessments, although it has not been validated to guide adjuvant treatment decisions.

In the postoperative setting, cfDNA and ctDNA may eventually serve as adjuncts for MRD assessment and recurrence monitoring. Early TET-specific studies suggest that cfDNA correlates with metastatic burden and that ctDNA can detect tumor-associated alterations during longitudinal follow-up, but current evidence is insufficient to guide routine postoperative management [90,91,93]. These findings raise the possibility of risk-adapted surveillance or treatment, but current evidence is insufficient to guide routine postoperative management.

## 9. Barriers to Individualized Therapy in Rare Thymic Epithelial Tumors

**Figure 3:**
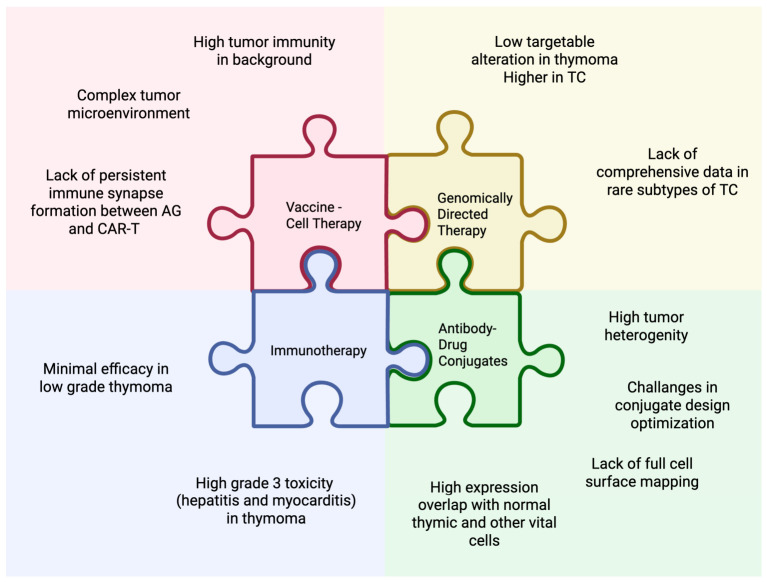
Treatment for thymic epithelial tumors: current landscapes and emerging therapeutic challenges. This conceptual framework summarizes the main biological and clinical challenges limiting individualized treatment strategies in thymic epithelial tumors. Genomically directed therapy is constrained by the low frequency of targetable alterations in thymoma, higher but heterogeneous genomic alterations in thymic carcinoma, and limited comprehensive data in rare thymic carcinoma subtypes. Immunotherapy is limited by modest efficacy in low-grade thymoma and a high risk of grade ≥3 immune-related toxicities, particularly hepatitis and myocarditis. Vaccine and cellular therapy development is challenged by the complex tumor microenvironment, high baseline tumor-associated immunity, and limited persistence of antigen–CAR-T immune synapses. Antibody–drug conjugate development is restricted by tumor heterogeneity, incomplete cell-surface target mapping, challenges in conjugate design optimization, and overlap of target expression with normal thymic or other vital tissues. AG, antigen; CAR-T, chimeric antigen receptor T-cell therapy; TC, thymic carcinoma. Created in BioRender. Canaslan, K. (2026). https://BioRender.com/48dw9sd accessed on 19 July 2026.

When evaluating the impact of actionable genomic alterations on patient survival in thymic epithelial tumors, the absence of high-level evidence is primarily driven by the unique biological and clinical challenges associated with these rare malignancies (Figure 3). Comprehensive genomic profiling identifies potentially relevant alterations more frequently in TC than in thymoma [22]. However, alterations with established predictive value and a clinically validated matched therapy remain uncommon, particularly in thymoma [102].

In the prospective MASTER trial, comprehensive multi-omics profiling enabled molecularly informed treatment recommendations for 86% of patients with advanced TETs [103]. However, this figure should not be interpreted as the prevalence of validated actionable drivers. Recommendations included off-label and investigational therapies supported by different levels of molecular and clinical evidence. Only 45% of patients for whom a recommendation was made ultimately received the proposed treatment, highlighting the gap between theoretical molecular actionability and treatment implementation. Among evaluable patients who received molecularly informed therapy, the reported ORR was 23% and the DCR was 62% [16]. Precision oncology programs match patients to a wide variety of off-label drugs depending on their unique tumor profile. Evaluating a single survival metric, such as overall survival, across a molecularly heterogeneous cohort treated with dozens of different substances is statistically challenging and prevents traditional meta-analyses [16]. Similarly, a clinicogenomic study demonstrated that 60% of patients with metastatic TETs harbored molecular alterations with known or potential therapeutic implications. In their cohort, 54% of patients subsequently received at least one targeted therapy. For patients whose treatment was eventually stopped due to disease progression rather than toxicity, the mean time to progression was 9.9 months. However, nearly half of the treated patients discontinued targeted therapy early because of toxicity [22].

Furthermore, because of the rarity of the disease, conducting large-scale randomized controlled trials comparing targeted therapy against standard chemotherapy requires years of trial accrual. While the literature lacks level 1 evidence proving an OS benefit, the MATCH trial showed that matching patients to their actionable genomic alterations can slow disease progression and provide a therapeutic advantage [16]. The integration of precision medicine into the management of TETs faces significant economic and logistical hurdles, particularly in developing countries. While NGS is essential for identifying actionable targets, the high cost of whole-genome sequencing (WGS), panel-based genomic testing and the complex computational demands required for analysis remain major limitations [21]. The cost of the therapies themselves represents a formidable barrier to individualized care. Data from the prospective trial highlight a stark gap; although molecularly informed therapy was recommended for 86% of advanced TET patients, it was actually administered to only 45%, frequently due to the challenges of accessing expensive, off-label drugs [16]. Consequently, while genomic profiling offers a theoretical blueprint for personalized treatment, the reality of therapeutic delivery is strictly constrained by a patient’s socioeconomic status and the healthcare infrastructure of their country.

Because organizing large-scale, randomized phase III trials is virtually impossible in this population, there are currently no targeted therapies or immunotherapies specifically approved by the FDA or EMA for TETs. Later-line use of agents such as sunitinib, lenvatinib, and pembrolizumab therefore often depends on guideline recommendations, off-label access, and local reimbursement policies.

## 10. Future Directions

The management of TETs is often complicated by a lack of specialized expertise in histological diagnosis and subtyping, which is critical given the significant divergence in systemic treatment strategies between thymoma and thymic carcinoma. To address this diagnostic ambiguity and the necessity for more precise, biologically driven therapy, integrating advanced diagnostic modalities is essential.

Artificial intelligence (AI) and machine learning methods may facilitate this multimodal integration [13]. CT-based radiomic features have differentiated thymoma from the more aggressive TC, with an area under the curve (AUC) of 0.882, increasing to 0.921 when combined with patient sex [104]. Furthermore, machine learning applied to non-contrast CT scans has successfully predicted WHO subtypes with an AUC of 0.935 [105]. Similarly, utilizing CT-derived radiomic features to train machine learning classifiers has yielded good discriminatory performance for predicting histologic subtypes (AUC 0.876) and early versus advanced TNM stages (AUC 0.838) [106]. However, the radiomics model yielded poor predictive performance for detecting the presence of myasthenia gravis (AUC 0.639) [106]. These findings demonstrate the feasibility of noninvasive tumor phenotyping but do not establish an ability to predict benefit from a specific treatment.

AI-assisted digital pathology may provide complementary information. Zullo et al. developed a multiple-instance learning model based on digitized hematoxylin–eosin–saffron-stained whole-slide images from the French RYTHMIC network [107]. This pathomics model successfully classified the major WHO histological subtypes (A, AB, B1, B2, B3, C), achieving a mean AUC of 0.94 during internal validation and 0.89 in a prospective test cohort. The model identified regions with varying proportions of epithelial cells and lymphocytes, demonstrating the potential of AI-assisted digital pathology to characterize TET heterogeneity and support histologic classification [107]. Computational analysis of multi-omics data has also predicted candidate neoantigens associated with CYLD and KMT2C alterations, providing a potential basis for personalized vaccines or adoptive cellular therapies [108]. Future studies should move beyond diagnostic accuracy and determine whether multimodal models improve biopsy selection, treatment-response prediction, therapeutic stratification, and clinical outcomes.

## 11. Conclusions

TETs are biologically heterogeneous malignancies that remain challenging candidates for individualized therapy. Although TC is enriched for potentially relevant alterations such as TP53, CDKN2A/B, KIT, CYLD, BAP1, and PI3K/AKT/mTOR pathway abnormalities, most molecular findings remain prognostic or biologically informative rather than directly actionable. To date, antiangiogenic strategies, particularly lenvatinib, sunitinib, and ramucirumab-based chemotherapy, provide the strongest targeted-therapy evidence in TC. In contrast, approaches targeting mTOR, EGFR, KIT, IGF-1R, HDAC, and DNA damage response pathways have shown limited or highly selected activity. ICIs may induce durable responses in selected patients with TC, but their use is constrained by severe immune-related toxicity, including myocarditis, particularly in thymoma. ctDNA monitoring, ADCs, vaccines, and cellular therapies are promising but remain investigational. Progress in this rare disease will require molecularly annotated international trials, systematic evaluation of matched therapy, validated liquid biopsy approaches, surface-target discovery, and equitable access to genomic testing and targeted treatments. Emerging precision technologies may also support perioperative decision-making, response assessment after induction therapy, and postoperative surveillance, although prospective validation is required.

## Figures and Tables

**Table 2:** Immune and antibody-based therapies with clinical evaluation or active TET-directed investigation.

Agent/Strategy	Target	TET Population	Evidence Status	Efficacy	Key Limitation
Pembrolizumab	PD-1	Recurrent/refractory TC	Phase II [53]	ORR 22.5%	Grade ≥ 3 treatment-related AE 13%, grade ≥ 3 irAE 15% (including myocarditis)
Pembrolizumab + lenvatinib	PD-1 + VEGFR/FGFR multikinase inhibition	Pretreated B3 thymoma and thymic carcinoma	Phase II (PECATI) [59]	ORR 23.3%, DCR 93.0%, 5-month PFS 88.4%, median PFS 14.9 months	Grade ≥ 3 treatment-related AE in 37% and grade ≥ 3 irAEs in 14%
Nivolumab	PD-1	Recurrent TC	Phase II (PRIMER) [61]	RR 0%, median PFS 3.8 months	Early termination
Nivolumab	PD-1	Recurrent/refractory B3 thymoma and TC	Phase II (NIVOTHYM) [62]	ORR 12% and median PFS 6.0 months	ORR 12% grade ≥ 3 AE 57%
Nivolumab + ipilimumab	PD-1 + CTLA-4	B3 thymoma and thymic carcinoma	Phase II (NIVOTHYM Cohort 2) [54]	ORR 17.7%, DCR 60.8%, median PFS 3.2 months, 6-month PFS rate 21.6%	Grade ≥ 3 treatment-related AEs in 29% (including myocarditis and colitis)
Avelumab	PD-L1	Advanced thymoma	Phase I [63]	29% partial response	All responders developed irAEs
Avelumab + axitinib	PD-L1 + VEGFR	Pretreated B3 thymoma and thymic carcinoma	Phase II (CAVEATT) [60]	ORR 34%, DCR 91%, median PFS 7.5 months, median OS 26.6 months	12% severe new-onset irAEs (polymyositis, pneumonitis)
Atezolizumab	PD-L1	Thymoma/TET cohorts	Phase II basket trial [64]	ORR 38.5% in thymoma, 8.3% in TC	35.7% serious AEs in thymoma
Atezolizumab + carboplatin + paclitaxel	PD-L1 + chemotherapy	Newly diagnosed advanced TC	Phase II (MARBLE) [65]	ORR 56%, median PFS 9.6 months	Grade ≥ 3 AEs in 75% and high irAE rate (66.7%)
WT1 peptide vaccine	WT1	Advanced thymoma and thymic carcinoma	Phase II [66]	ORR 0%, disease stabilization 75%	Pure red cell aplasia and myasthenia gravis were observed after prolonged administration
Sacituzumab govitecan	Trop2	Advanced thymoma and thymic carcinoma after prior systemic therapy	Phase II ongoing [67]	Not mature	Myelosuppression and diarrhea are expected based on class effect
Anetumab ravtansine	Mesothelin	Mesothelin-expressing tumors including thymic carcinoma	Phase Ib [68]	Study completed, not reported	Not established
CAR-T/engineered cell therapy	Variable surface targets	No established TET-specific product		No validated clinical efficacy in TETs	Not established

Abbreviations: AE, adverse event; CAR-T, chimeric antigen receptor T-cell therapy; DCR, disease control rate; FGFR, fibroblast growth factor receptor; irAE, immune-related adverse event; ORR, objective response rate; OS, overall survival; PD-1, programmed death-1; PD-L1, programmed death ligand-1; PFS, progression-free survival; TC, thymic carcinoma; TET, thymic epithelial tumor; TROP2, trophoblast cell-surface antigen 2; VEGFR, vascular endothelial growth factor receptor; WT1, Wilms tumor 1.

## Data Availability

No new data were created or analyzed in this study. Data sharing is not applicable to this article.
